# Use of community-based interventions to promote family planning use among pastoralist women in Ethiopia: cluster randomized controlled trial

**DOI:** 10.1186/s12905-021-01434-x

**Published:** 2021-08-18

**Authors:** Mussie Alemayehu, Araya Abrha Medhanyie, Elizabeth Reed, Afework Mulugeta Bezabih

**Affiliations:** 1 School of Public Health, Mekelle University, College of Health Sciences, Mekelle, Ethiopia; 2 Graduate School of Public Health, San Diego State University, San Diego, USA

**Keywords:** Afar, Family planning, Pastoralist, Clustered randomized controlled trial, Male involvement in family planning, Women’s education in family planning

## Abstract

**Background:**

Afar region is one of the pastoralist dominated regions in Ethiopia. The region is characterized by a low contraceptive prevalence rate (CPR) of 5.4%. Lack of awareness of contraceptive use, husband objection and religious barriers are attributed to low CPR in the region. This study assessed the effect of community-based interventions for promoting family planning (FP) use among pastoralist communities in Ethiopia.

**Methods:**

The study design was a three-arm, parallel, clustered randomized controlled trial (CRT). The three study arms were: (1) male involvement in family planning (FP) education; (2) women’s education on FP; and (3) control. A total of 33 clusters were randomized and allocated with a one-to-one ratio. Intervention components included (1) health education on FP to married women and men by faema leaders (a traditional community-based structure that serves as a social support group); (2) video-assisted message on FP; and (3) assisting the faema leader using health workers and health extension workers (HEWs). The intervention was given for a total of nine months. FP use and intentions were measured as outcome variables. In addition, a cluster-level summary considering a cluster effect analysis was performed. The result was presented with t.test, adjusted risks and its 95% confidence interval (CI).

**Results:**

The proportion of FP use among the arms was 34% in the male involvement in FP education, 17.5% in women’s education on FP and 4.3% in the control. There was a positive change in the proportion of FP use in the male involvement in FP education and women’s education on FP arms with absolute risk (AR) of 0.29 (95% CI, 0.23,0.34) and 0.13 (95% CI,0.08,0.17), respectively, as compared to the control arm. Similarly, married women in the male involvement in FP education and women’s education on FP arms had 3.4 (2.48, 4.91) and 2.1 (1.50, 2.95) higher intention to use FP, respectively, as compared with the control arm.

**Conclusion:**

The present study suggests that in such male-dominated pastoralist communities with more considerable religious barriers, the community-based health education mainly targeting males appears to be a promising strategy for improving FP use and intention to use FP.

***Trial registration*:**

ClinicalTrials.gov, NCT03450564, March, 2018.

**Supplementary Information:**

The online version contains supplementary material available at 10.1186/s12905-021-01434-x.

## Background

Globally, the level of fertility is expected to continue declining. The global level of fertility is expected to reach 2.2 live births per woman in 2050 and 1.9 in 2100, with 3.1 and 2.1 in Sub-Saharan Africa (SSA), on the respective periods. As modern contraceptive use rate increases fertility rate declines. In 2019, 49% of women in reproductive age were using a modern method of contraception, an increase from 42% in 1990, where it went from 13 to 29% in SSA [1]. In developing countries, including Ethiopia, maternal and child morbidity and mortality have remained high over the past decades, despite international efforts to increase FP use and decrease these deaths [2]. Such a burden can be averted by increasing effective family planning (FP) use as a means to decrease unintended pregnancy and its consequences, thereby reducing maternal and newborn morbidity and mortality [2, 3]. However, globally in 2018, nearly 214 million women of reproductive age in developing countries who want to avoid pregnancy were not using a modern contraceptive method. SSA, including Ethiopia, accounts for the highest unmet need for FP among women of reproductive age group, with 24.2% of women meeting the definition for having unmet need for FP [4]. And, such a figure including the measurement of FP would worsen in the pastoralist community. Despite this low rate, the globe has targeted to access and use FP for an additional 120 million SSA women in the year 2020 [5]. Indeed, FP promotes inclusive societies by addressing the needs of disadvantaged populations like pastoralist communities [6].

About 12 million Ethiopian pursue pastoralism as their way of life. They live in the most inaccessible and remotest parts of the country. As a result, they are underserved in health, social services and economic infrastructures [7]. Afar region is one of the pastoralist dominated regions in Ethiopia. Ethiopia had set a goal to achieve a contraceptive prevalence rate (CPR) of 55% and total fertility rate (TFR) of 3 by the year 2020 [8], despite the CPR of 41% in 2019 [9] and TFR of 4.6 in 2016 [10]. However, FP use among pastoralist communities’ such as those in the Afar region is lower than the national average. The region’s FP utilization is low characterized by CPR ranges from 5.4 to 12.7% [10, 11], TFR of 5.5 and unmet need for FP of 17.2% (12.9 for spacing and 4.3% for limiting) [10]. The lack of awareness//knowledge of FP methods among women, religious barriers to FP use and other social norms are often cited as the leading causes of low FP use and large family size in the region [12, 13]. In addition, women often report strong opposition from their husbands to FP use coupled with their low decision-making power compared to men, limiting their right to access the FP service, as factors contributing to low FP use in the region.

Furthermore, the Health Extension Program (HEP), which is believed to be one of Ethiopia’s best programs in disease prevention, promoting good health and the use of FP service, is less practiced in the Afar region [14]. Also, the women development army (WDA) established to strengthen the HEP in creating awareness, increase health-seeking behavior and build a community sense of ownership has not been yet established in contrast to the agrarian regions of the country [15]. Moreover, there is a dearth of evidence on quantifying the effect of community-based interventions for promoting the number of FP users and intention among married women in the pastoralist communities.

During 2014–2018, the Reproductive, Maternal and Newborn Health Innovation Fund (RIF) launched a project aimed to enhance the maternal and child health services (antenatal care, institutional delivery and postnatal care) in Ethiopia, including FP of the pastoralist communities [12]. Research has indicated that male involvement in FP education and educating women on FP is critical to increasing FP use in the Afar region [16, 17]. Along with, studies done elsewhere (pastoralist and non-pastoralist communities) showed an increment in the rate of FP use as a result of different community-based interventions. For instance, a study done in different districts of India had applied various community-based intervention; including organizing group meetings, providing trainings to rural providers and community leaders [18] creating awareness and encouraging inter-spousal communication to enhance FP use [19]. Indeed, male involvement, one health approach, a migratory route of container clinic, mobile clinic and building maternity waiting home are some of the interventions that contributed to enhanced maternal, newborn and child health (MNCH) in pastoralist communities [20–23]. Notably, a study in a pastoralist community of Kenya showed improvement in the MNCH service utilization using male involvement [22]. Moreover, other evidence shows that as the women’s decision-making capacity increases, the proportion of women who use FP also increases [24]. Women’s decision-making regarding FP can be further enhanced through involving a male partner in FP service [19, 20]. Therefore, community-based interventions can be applied at the community level to improve the low rate of FP use/practices in the pastoralist communities.

Hence, using community-based interventions focusing on approaching the target groups or communities using their own community members could be an excellent strategy to educate women and men. In Afar, there is “faema” a traditional community-based structure in the pastoralist community that serves as a social support group. It has a long history and high community acceptance and has a separate structure for males and females [16]. The authors of the study had believed that the faema structure could be a good strategy for enhancing FP use and intention to use FP among the pastoralist communities. So, one rationale of the study was assessing if a structured health education provision about FP given by faema would be more effective than the unstructured educational approach to increase FP use and intention to use FP. It was also hypothesized that having male involvement in FP education and educating women on FP as part of community-based interventions could increase the number of married women who use and intend to use FP in the target groups. A rigorous method (cluster randomized controlled trial (CRT)) was employed to achieve the goal as it was believed to be practically feasible and prevents contamination of the disseminated information at the ground [25]. The main aim of the study was to evaluate the effect of community-based interventions using CRT on FP use and intention among married women in the pastoralist communities of the Afar region, Ethiopia.

## Methods

### Study design

A three-arm, parallel, clustered randomized controlled trial (CRT) (male involvement in FP education, women’s education on FP and control) was used. One-to-one ratio allocation of the intervention with a control arm was employed to assess the effect of community-based interventions to promote FP use and intention to use among the pastoralist married women. A repeated cross-sectional (cross-sectional at baseline and end-line) type of data was used to collect the intended information from the married women. It contains baseline and end-line data collection with a nine-month duration to assess change over time in the study outcomes.

### Participants

The cluster was created based on geographic boundaries. The clusters included in the study had at least 30 households with married women. They were chosen with natural borders and has enough distance (20–40 km) from adjacent clusters to reduce the risk of contamination information. Inclusion criteria for women participants included being married and residing in a given cluster in the Afar region. In addition, women were excluded from the study if they reported infertility or a severe illness.

### Study setting and period

The study was conducted in the Afar region which is one of the ten regional states of Ethiopia. It is characterized by presence pastoralist and Muslim dominated communities and is composed of five zones, 32 woredas (districts), five town administrations and 404 kebeles (lowest administrative unit). It is home to an estimated population of 1,816,304 (with 799,174 (44%) females)) [26]. Majority of the population reside in rural areas and are pastoralists or agro-pastoralist whose religion is dominantly Muslim [13]. The region is characterized by high rates of early marriage, which is mainly influenced by parental decisions and a high prevalence of early pregnancy and delivery. It is also known for a significantly high illiteracy rate (75.6%), low CPR (5.4%) [11] and high unmet need for FP (17.2%) [10]. Furthermore, a clan-based system favoring large family sizes [16], wide spread practice of being in a polygamous union of marriage and a high burden of work among women are a peculiar characteristics of the people [27]. Poor access to health care facilities forces women in the region to travel long distances [16] and often demand accompany of family members [28].

For this study, three districts (Mille, Afambo and Kori) were included in the intervention. These three districts are found with a radius of 150 km away from the capital city of the region, Semara. These districts have relatively better infrastructures (road accessibility and transportation services) than the other parts of the region. In 2018, majority of the population (76–86%) in these districts were unable to read and write where as in the Afar region it ranges from 75.6 to 89.7% [10, 13]. More than nine-in-ten of the inhabitants were Muslim followers in religion, where as it was 98.8% in Afar region [13]. Nearly nine-in-ten of the inhabitants in the selected three districts and Afar region were pastoralist/agro-pastoralist in occupation [11]. The FP use was 3.4% in Kori, 3.7% in Afambo and 5.7% in Mille, where as it ranges from 5.4 to 12.7% in Afar region [10, 11]. According to the Afar Health Bureau report, the region has one regional hospital, six zonal hospitals, one non-govermental hospital (NGO), 78 health centers and 379 health posts [29]. Moreover, the selected districts have the following health service. In Mille there are (one NGO hospital, three health centers and seven health posts), Afambo (two health centers and five health posts) and Kori (two health centers and five health posts). The health centers and health posts provide maternal and child health service including FP to the pastoralist communities. Likewise, the majority part of the region’s districts has a similar number of health centers and health posts [30]. The intervention was carried out for nine months, January to September 2018.

### Community-based interventions

The interventions were targeted at the cluster level. The following community-based interventions were applied; (1) male involvement in FP education, and (2) women’s education on FP. Each intervention was compared with the control arm in terms of FP use and intention to use. While the targets for male involvement in FP education arm were both married women and men, the targets for women education on FP arm were only married women. The health education in the male involvement in FP education arm was given separately for married women and men. It was designed with the principle of approaching the community with their own community members (faema leaders). The faema structure was assessed to be preferable to provide the intervention in areas like Afar region where the health extension programs (HEP) were not strengthened compared with the agrarian regions of Ethiopia [16].

HEWs are frontline health workers adopted by the government of Ethiopia (GOE) to achieve universal coverage of primary health care among its rural population by 2009. They served as a significant source of health information, including FP [14]. Never the less, HEWs structure has not been strengthened in Afar region. Furthermore, the women development army (WDA) is a structure at the community level, which was evident in the agrarian regions to strengthen the HEP in improving maternal and child health, including FP utilization. However, it has not been yet established in the region [15]. Hence, to enhance FP use among the pastoralist married women, the following approaches were applied to reach the target groups; (1) provision of health (FP) education to married women and men by faema leaders; (2) video-assisted messages on FP from role model people (married women who started to use FP, district’s FP experts, male who actively involved in FP service and religious leader), and (3) assisting the faema leaders using HEWs and health workers in their own communities.

**Fig. 1:**
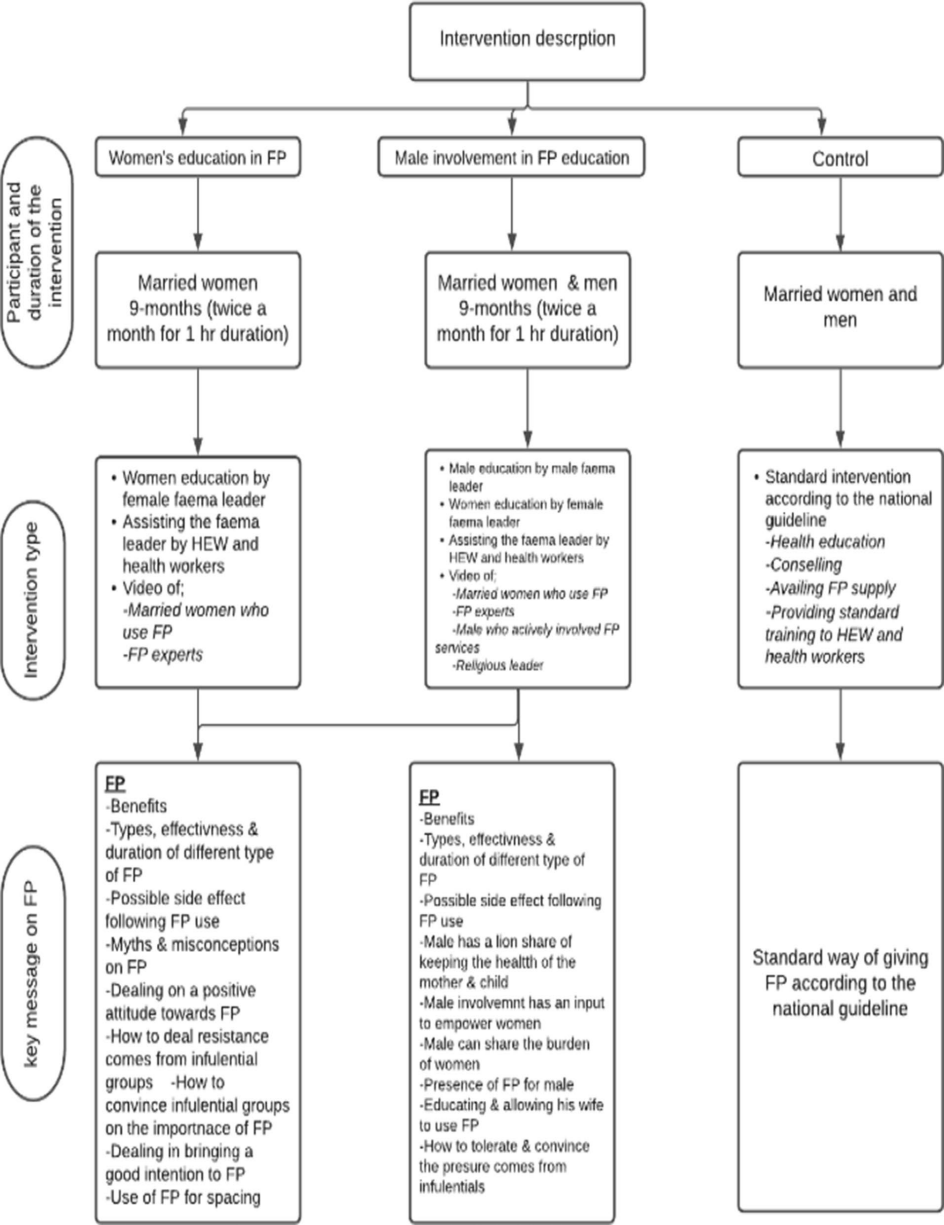
Type of family planning education per arms among pastoralist community Afar region, Ethiopia, 2018

Before the FP message was provided to the community, a tailored message which is highly acceptable in the community was discussed. Accordingly, the emphasis of the message was given on the purpose of FP for maintaining spacing between children than limiting the number of children. In Afar, it was challenging to discuss and raise an issue about FP despite having the highest maternal, neonatal and child mortality rates in the region. After an extensive search to identify the barriers of reproductive, maternal, neonatal and child health (RMNCH) in the region, it was found that FP coverage was the lowest indicator with 5.4% [11]. Along with this fact, the lack of awareness, husband objection and religious influence were potent barriers for not using/ exercising FP [13]. Accordingly, FP messages were developed, qualitative data collected from the key informant on the applicability of the messages and a draft FP messages was prepared by experts (reproductive health specialists and health education and promotion experts). The developed messages were tested with the community representatives (married women, men, FP experts and religious leaders) and collected constructive comments to improve the draft FP message. Finally, a consensus was reached on FP education to the community to emphasize the purpose of FP for spacing between children’s than limiting the number of children to better convey the message through community members (faema leaders in educating the married women and men). The intervention was guided using an integrated behavioral model (IBM) [31]. A detailed description of these community-based interventions based on the type of arm is described below (Fig. 1).

**(1) Male involvement in FP education arm:** In this arm, the following interventions were provided; (1) provision of health (FP) education to both married women and men by female and male faema leaders, respectively; (2) video-assisted message on FP (from married women who started to use FP, district’s FP experts, male who actively involved in FP service and religious leader); and (3) assisting the faema leaders using HEW and health workers. A detailed description of each intervention are described below.

(**A) Health (FP) Education by Faema leaders:** This targeted both married women and men in the cluster. Male faema leaders were used to approach men while the female faema leaders approached married women. Two female and two male faema leaders from each cluster were trained for three days. In the beginning, intensive training was given for the faema leaders on a different aspect of FP. The training for female faema leaders included a detailed description of FP experience of Muslim dominated countries and its relation with reducing total fertility rate (TFR) and maternal mortality [32], and on how to start to positively influence the neighbors in their catchment to use FP and on details of FP. The contents covered under details of FP included definition of FP, types of FP, purpose of FP, effectiveness and duration of prevention from unwanted pregnancy. It also included sessions that covered myths and misconception on FP and its side effects, how to overcome the pressure/ resistance that comes from influential groups (husband, neighbors, clan and religious leaders) and being a role model by starting to use FP.

After the training, with the mobilization of the faema leaders, a regular meeting on FP was being organized at center of the cluster. The meeting was held twice a month in the afternoons with a one-hour duration. A regular schedule was prepared to ensure that health education messages were uniform across clusters in each session. Notably, a logbook or registration book was prepared to follow the progress of the intervention. It contained personal details (such as names, sex and age) of the participants and type of FP topic discussed in each health education session. The logbook was checked by the research team once a month.

Similar training and meeting modalities (schedule, duration, filing/documentation, etc.) were used for dealing with both male and female faema leaders in the male involvement arm except that in the males group the content gave more focus was given on encouraging men/husbands to actively engage on FP. The males group further emphasized promoting spousal communication, allowing one’s wife to use FP, accompanying her to health facility for FP use, reminding her of the schedule of taking FP, participating in choosing the type of FP, providing her financial support and helping her in domestic work.

(**B**) **Video-assisted message on FP**: Recorded videos on FP from married women who started to use FP, district’s FP experts, married men who actively involved in FP service and religious leaders were prepared. The video message from married women dealt with the life experience related to FP (its process of using FP, benefit, challenges faced and actions taken). The video message from the district’s FP experts discussed the benefits of practicing FP, types of FP, possible side effects of FP, management of FP side effects and availability of FP in health facilities. The experience of a husband who was actively involved in FP services such as allowing his wife to use FP, accompanying her to health facility, participating in choosing the type of FP, providing financial support and helping her in domestic activity was recorded and used to teach the men under the male involvement in FP education arm.

Also, a video-recorded message from a religious leader was delivered for the men in the cluster. Earlier, the importance of FP for maintaining spacing between one’s children was discussed with the religious leaders, and a consensus was reached. After they agreed on the importance of FP, ensuring harmony/alignment of the message on FP with Islamic religion, and identifying type of FP which did not contradict their religion, the video-assisted message was recorded and disseminated.

The recorded video messages of the different groups (women who started to use FP, district’s FP experts, etc.) were uploaded to tablet smartphones. The tablets, with their accessories, were given to the faema leaders (male and female) to help them disseminate the FP messages while teaching the community under their respective clusters. It was given for a total of six months. Training on operating, delivering, and teaching the recorded video messages was demonstrated and re-demonstrated by the faema leaders. All the FP messages were prepared in the local language “Afarrigna”.

(**C**) **Assisting the faema leader using HEWs and health workers**. In the beginning, the HEWs and health workers working at FP were trained on FP. The health workers working at FP took special training (making the health facility ready for FP service, availing method mix timely, managing side effect following FP use and counselling married women on FP use based on informed consent). Furthermore, the HEWs were trained to assist faema leaders (male and female) during FP health education programs and provide house-to-house counselling to voluntary married women to use FP services. They also facilitate referral linkage for married women who prefer to use FP, including long-acting FP, to the health centers.

(**2) Women’s education on FP use arm:** Interventions provided under this arm were; (1) health (FP) education to married women (only—excluding their husbands) by female faema leader; (2) video-assisted message on FP and (3) assisting the faema leader using HEW and health workers. This arm type was similar to male involvement in the FP education arm except for the points described below. Firstly, the FP health education by faema leaders targeted only married women in the cluster and did not include male as described in the male involvement in the FP education arm. Secondly, the video-assisted message on FP was recorded only from married women who started to use FP and the district’s FP expert to teach the married women on (benefit of FP, type of FP, possible side effect, management of side effect and availability of FP in health facilities). It should be noted that the content of the video-recorded message from married women who started to use FP and the district’s FP expert was similar to the male involvement in the FP education arm. However, this arm, it did not use a recorded video message from married men who had been actively involved in FP service. Likewise, messages from religious leaders were not included unlike in the male involvement in FP education arm. Thirdly, on the assistance of the faema leader using HEW and health workers, the activities of health workers on (making the health facility ready for FP service, availing method mix timely, how to manage side effect following FP use and counselling married women on FP use based on informed consent) was similar with the male involvement in FP education arm except with the target/support of HEWs. The HEWs’ target in this arm was to support only the female faema leaders as there were no male faema leaders. However, there was no difference in the activities (assisting faema leaders during FP health education programs and providing house-to-house counselling to voluntary married women to use FP services and facilitating referral linkage for married women who prefer to use FP) with the male involvement in FP education.

(**3) Control arm:** In this arm, the control communities did not receive the above mentioned interventions which were applied to the first two arms, but received just the standard intervention according to the national guideline, which includes assigning health professionals at health facilities, availing FP supply and providing standard FP training to HEW and health workers. It should be noted that there was no difference in the FP supply among the control and intervention arms because the government is responsible for availing it (Fig. 1).

### Measurement of the outcome variables

The purpose of this study was to evaluate the effect of community-based interventions (male involvement in FP education and women’s education on FP) compared to the control arm at the cluster level to increase rate of FP use and intention to use. FP indicators were measured based on the number of married women’s who use and intend to use FP. The primary outcome variable was actual practice or use of modern FP method (s) with the question of “*Are you or your partner currently doing something or using any method to delay or prevent getting pregnant?*”. Moreover, the type of modern FP alternative (pill, Depo-Provera, condom, Jadelle, Implanon, IUCD, etc.) used by the woman or her husband was collected.

Intention to use FP was taken as a secondary outcome variable. A total of eight items that ranged from the lowest level (*At this moment, I can list some benefits of FP use and I would gain if I use it*) to the highest level of intention to use FP (*It is expected that women in our community should use FP and so do I*) were used to initiate responses. The responses ranged from 1 (uncertain/disagree) to 3 (certain/agree). The responses were summed up to form a continuous variable. It was categorized based on the response of married women mean value into “*low intention to use FP*” and “*high intention to use FP*” for those married women who scored mean and below mean and above mean, respectively.

In addition to the primary and secondary outcomes, the following variables were collected. First, the community responsibility was collected to describe spouse’s responsibility either as a clan, religious and faema leader. In line with this, being a faema leader for married women also included as a community responsibility. Second, along with a positive/yes response by married women for FP’s current use, the husband’s knowledge (whether he is aware or not) about the current use of FP and the type of support obtained from him were included in the study. Types of support which were checked whether they were provided by the husband or not included accompanying one’s partner to the health facility, reminding her of the schedule for taking the chosen FP alternative, participating in choosing the appropriate type of FP alternative and helping her in domestic activity.

### Study design, sample size determination, and sampling procedure

The sample size was calculated using the literature of Richard and Lawrence [33] to determine the number of clusters required to detect a difference among different arms. The sample size calculation considers the current FP use in Afar region of 11.6% [10] expected changes to be acquired following the intervention of 20%, 90% power, 95% confidence interval, considering the intracluster correlation of ρ = 0.05, adjusting for non-response of the individual in a household of 20% and a design effect of 2.2. Considering an equal number of clusters and cluster sample size, the final sample size was 33 clusters where each cluster had 27 married women. A systematic sampling technique was used to select 27 married women from one cluster. A sampling fraction was calculated based on the total number of married women in the cluster. A random start number was selected to identify the first married woman in the clockwise direction. Hence, nine clusters (five in male involvement in FP education and four in women’s education on FP) of Afambo, seven clusters (five in male involvement in FP education and two in women’s education on FP) of Mille and six clusters (two in male involvement in FP education and four in women’s education on FP) of Kori were included in the intervention. The same sampling procedure was used to collect the follow-up data for the baseline and end-line data.

### Randomization

A cluster randomized controlled trial, parallel-group study design with three arms was implemented. Simple randomization was used to allocate the number of clusters into three arms (male involvement in FP education, women’s education on FP and control) using a computer-generated random number. A different person’s allocation was done other than the principal investigator to avoid bias during the process. In addition, clusters were randomized into two intervention arms and control conditions before the initiation of enrollment.

### Data collection tools and procedures

A questionnaire was developed for this study and attached as an additional file. The tool was developed by reviewing different literatures on previous findings that explore barriers and facilitators to Reproductive Maternal Neonatal Health (RMNH) services including FP and Ethiopian Demographic Health Survey (EDHS) report [10, 11, 13]. The developed tool was piloted in 10% of the sample and exposed to a reliability and validity test. It was done to assess the consistency of items in each construct (Cronbach’s Alpha > 0.7). Besides, exploratory and confirmatory factor analysis was done [34]. After all necessary corrections made followed the piloted test, the tool was pretested in 5% of the sample to assure wording, skip pattern and determine the time allotted to complete one interview. A repeated cross-sectional type of follow-up data (baseline and end-line data) was used to collect the data as there was a fear of high migration among the pastoralist community.

The secondary outcome variable (intention to use of FP) was constructed of eight items that had Cronbach alpha of 0.93, explained 87.7% of the variance with Kaiser–Meyer–Olkin **(**KMO) of 0.84 and Bartlett’s Test of Sphericity of chi-square (*df*) value of 9248 [28] and significant at a *p*-value of < 0.001 [34]. Six clinical nurse data collectors and two supervisors were used to collecting the data after training on the items and how to use mobile-based applications. They were recruited outside the study/intervention areas and assigned to a different cluster of given districts. The baseline and end-line data were collected using an electronically smartphone-based application open data kit (ODK). Immediately after the data checked for its completeness, it was sent to the Mekelle University (MU) server, where the data were accessed and utilized by the research team.

### Data quality control

A reliable and valid tool was used to measure the outcome variables. The data collectors and supervisors were trained. The supervisors made regular supervision and follow-up. The data were collected using a friendly to use mobile-based application (ODK.) It ensures skip pattern, immediate scanning of the server’s collected tool, and avoids paper duplication costs.

### Data monitoring and safety

A team from Mekelle and Samara Universities and the Afar regional health bureau was established to monitor data safety. A volunteer married woman went to a health facility and counselled to use contraceptives based on her informed consent by health care providers. Furthermore, the research team effort to minimize the risk and maximize the benefit by following the provision of intervention using the protocol. Moreover, there was no risk of reporting following the provision of the community-based interventions.

### Statistical analyses

The data collected using ODK was exported to R software version 3.4.2 for analysis. Intention to treat analysis was used as a framework of analysis. All the analysis was used a 95% confidence interval (CI) and *p*-value < 0.05 to declared statistically significant. Since the number of clusters per arm was 11, a cluster-level summary was used [28] to compare the male involvement in FP education and women’s education on FP arms with the control arm. A separate cluster-level summary analysis was done to compare the control arm with the male involvement in FP education and women’s education on FP arms by considering the cluster effect.

It should be noted that the interest of this study was to compare the control arm separately with the intervention arms. Hence, no analysis was made between male involvement in FP education and women’s education on FP arms. Finally, the result of FP use and the intention was described with t-test, degree of freedom (*df*), *p*-value, the mean value of both groups (control and intervention) and adjusted risk with its 95% CI. Moreover, the prevalence ratio (the number of FP users at the end line divided into baseline data) was calculated. Finally, the odds ratio was calculated manually for FP use and intention from the absolute risk value to make our interpretation more understandable and informative [35].

### Any changes to the trial outcomes after the trial commenced

Even though efforts were made to strictly adhere to the originally established protocols, the following deviations were actually observed. Firstly, in the beginning, it was intended to provide the intervention for six months, however as the project life extended, the interventions were also extended for another three bringing the overall study period to nine months and, so, some arrangement of time were made in the provision of the interventions. Secondly, initially the data was planned to be analyzed using Generalized Estimating Equation (GEE) which allows for baseline or covariate adjustment in the final model. However, we could not run the model with GEE due to the limited number of clusters per arm (< 15). Hence, cluster-level summarizes was used to analyze the collected data and report the results [35].

## Results

### Participants and cluster flow

**Fig. 2:**
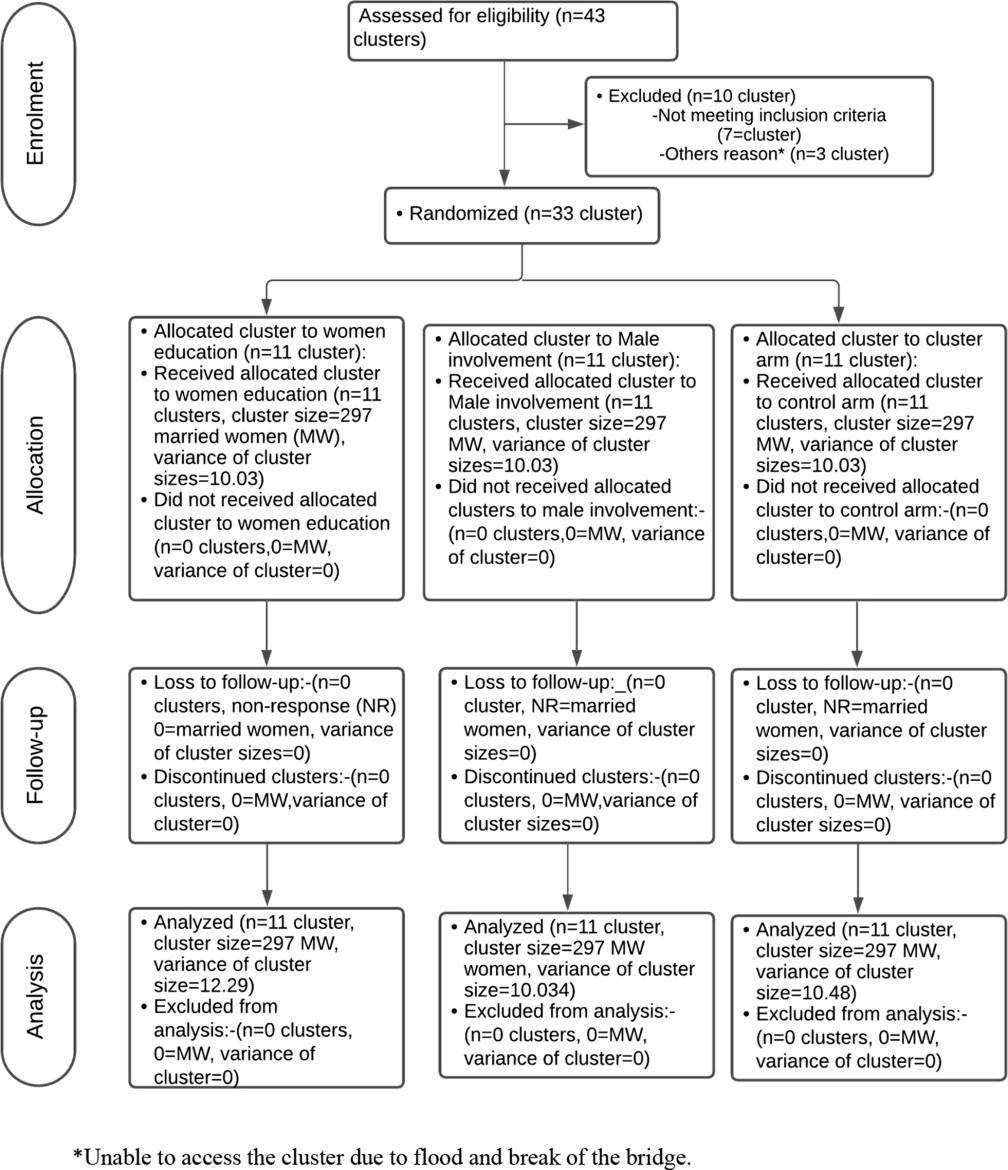
Participants and cluster flow of the trial among pastoralist community Afar region, Ethiopia, 2018

A total of 43 clusters were eligible for the study. Out of these, seven clusters did not fulfill the inclusion criteria and three clusters were physically inaccessible due to the breaking of the bridge as a result of heavy flooding. Hence, only 33 clusters were actually allocated to male involvement in FP education, women’s education on FP and control arms, with 11 clusters in each arm. Hence, the 33 clusters were followed and analyzed. There was no attrition among clusters. The variance of the cluster for FP use before the intervention and after the provision of the intervention in the male involvement in the FP education arm was 10.03 and 10.034, respectively (Fig. 2). The trial duration was nine months, starting in January 2018 and ending in September 2018.

### Baseline and end-line data information of respondents on selected variable’s

A total of 891 respondents (297 per each arm) participated in the baseline data. In the male involvement in FP education arm, the mean age of the respondents was 25.9 (± 6. 42) years, and their responses were analyzed as those who heard of FP method (s) previously were 269 (90.6%) and those who used FP method (s) before were 17 (5.7%). In the end line data, in the male involvement in the FP education arm, the mean age was 26.8 (± 6.10) years, and respondents who heard of FP methods were 279 (90.3%) and those who used FP method (s) were 102 (34.3%). The prevalence ratio of FP use was 1.8 in control, 3.7 in the women’s education on FP and 6 in the male involvement in FP education arms (Table 1).

**Table 1:** Before and after the provision of the intervention of selected variable characteristics of pastoralist married women per arms in Afar region, Ethiopia, 2018

Variables	Arms					
	Control		Women’s education on FP		Male involvement in FP education	
	Baseline data	End line data	Baseline data	End line data	Baseline data	End line data
	n (%)	n (%)	n (%)	n (%)	n (%)	n (%)
No of women	297	297	297	297	297	297
Mean (SD) age (years)	25.3 (6.4)	26.9 (6.7)	26.3 (6.8)	26.4 (6.7)	25.9 (6.4)	26.8 (6.1)
Heard of FP	252 (84.8)	240 (80.8)	265 (89.2)	265 (89)	269 (90.6)	279 (93.9)
Use of FP	7 (2.3)	13 (4.3)	14 (4.7)	52 (17.5)	17 (5.7)	102 (34.3)
Prevalence ratio (end line/baseline data) on FP use	1.8		3.7		6	

### Estimation of FP use among married women by study arms

The number of respondents (proportion) reporting use/yes of FP was 13 (0.48), 52 (1.93) and 102 (3.78) among the control, women’s education on FP and male involvement in FP education arms, respectively. By cluster, the mean (SD) of FP use was 0.043 (0.03) in control, 0.175 (0.05) in the women’s education on FP and 0.343 (0.09) in the male involvement in FP education arms (Table 2).

**Table 2:** Cluster level numbers and proportions of pastoralist married women reporting yes to overall FP use per arms following the provision of intervention, Afar region, Ethiopia, 2018

Clusters	Arms								
	Cluster size	Control (n = 11 clusters)		Cluster size	Women’s education on FP (n = 11 clusters)		Cluster size	Male involvement in FP education (n = 11 clusters)	
		# in clusters reporting yes	Cluster proportion reporting yes		# in clusters reporting yes	Cluster proportion reporting yes		# in clusters reporting yes	Cluster proportion reporting yes
1	27	1	0.04						
2	27	1	0.04						
3	27	2	0.07						
4	27	2	0.07						
5	27	0	0.00						
6	27	2	0.07						
7	27	1	0.04						
8	27	2	0.07						
9	27	2	0.07						
10	27	0	0.00						
11	27	0	0.00						
12				27	6	0.22			
13				27	4	0.15			
14				27	5	0.19			
15				27	7	0.26			
16				27	5	0.19			
17				27	7	0.26			
18				27	2	0.07			
19				27	5	0.19			
20				27	4	0.15			
21				27	4	0.15			
22				27	3	0.11			
23							27	10	0.37
24							27	11	0.41
25							27	13	0.48
26							27	9	0.33
27							27	14	0.52
28							27	9	0.33
29							27	7	0.26
30							27	8	0.30
31							27	6	0.22
32							27	7	0.26
33							27	8	0.30
Totals	297	13	0.48	297	52	1.93	297	102	3.78
Mean (SD)			0.043 (0.03)			0.175 (0.05)			0.343 (0.09)

### Characteristics of FP users by study arms in the end-line data

In the male involvement in the FP education arm, 9 (8.8%) of the FP users accounted for their husbands with community responsibility of the religious leader and it was 3 (5.8%) in the women’s education on the FP arm. Depo-Provera was the most common type of FP method (s) used by married women. The rate of utilization was 87 (85.3%) and 45 (86.5%) in the male involvement in FP education and women’s education on FP arms, respectively. In the male involvement in FP education, 88 (86.3%) of the participants disclosed to their husbands about using FP method(s) while this rate was 39 (75%) in the women’s education on FP arm. Information about the type of support obtained from their husbands indicated that 66 (76.7%) and 26 (66.7%) of the participants in the male involvement in FP education and women’s education on FP arms, respectively, reported that their husbands accompanied them to the health facility for FP use. Helping with domestic work by husbands was also reported as a type of support mentioned by 64 (74.4%) respondents in the male involvement in FP education and 26 (66.7%) in the women’s education on FP arms (Table 3).

**Table 3:** Description of FP users by selected variables per arms in Afar region, Ethiopia, 2018

Variables	Category	Arms		
		Control	Women’s education on FP	Male involvement in FP education
		Use of FP	Use of FP	Use of FP
		n (%)	n (%)	n (%)
Community responsibility of respondent’s husband	Religious leader	0 (0.0)	3 (5.8)	9 (8.8)
	Clan leader	3 (23.1)	5 (9.6)	10 (9.8)
	Faema leader*	1 (7.7)	5 (9.6)	17 (5.7)
Type of current FP use	Pill	1 (7.7)	4 (7.7)	6 (5.9)
	Depo-Provera	8 (61.5)	45 (86.5)	87 (85.3)
	Implanon	2 (15.4)	2 (3.8)	8 (7.8)
	Others**	2 (14.4)	1 (1.9)	1 (1)
Disclose to their husband for using FP currently	Yes	5 (38.4)	39 (75)	88 (86.3)
Get support from husband for use of FP	Yes	4 (80)	39 (100)	86 (97.7)
Type of support from husband on FP use	Accompany to a health facility	2 (50)	26 (66.7)	66 (76.7)
	Reminding the schedule	1 (25)	33 (84.6)	81 (94.2)
	Participate in choosing the type of FP	1 (25)	21 (53.8)	62 (72.1)
	Helping in domestic work	2 (50)	26 (66.7)	64 (74.4)

^*^Applicable for both married women and men; **Condom, Jadelle

### Effect of community-based interventions (male involvement in FP education and women’s education on FP) on FP use

The difference in FP use between the male involvement in FP education and control arms was 0.29 (95% confidence interval (CI): 0.23, 0.34). The FP use in the control arm was 4.3%, while it was 34.3% in the male involvement in FP education arm with an absolute risk (AR) increase of 29%; but this might be as little as 23% or as much as 34%. Similarly, the difference of FP use in women’s education on FP and control arm was 0.13 (95% CI: 0.08, 0.17). The proportion of FP use was 4.3% in the control arm, while it was 17.5% in the women’s education on FP arm with an AR increases of about 13%, but this might be as little as 8% or as much as 17%. Along with this, the baseline characteristics of FP use were not significantly different among the control arm with male involvement in FP education (t = 1.82, *p*-value = 0.0895) and women’s education on FP arm (t = 1.4, *p*-value = 0.1823) (Table 4).

**Table 4:** Estimated independent t-test coefficients to show the effect of male involvement in FP education, women’s education on FP versus control arm on FP use, Afar region, Ethiopia, 2018

Outcome	Mean value		t-test	*df*	*P*-value	Absolute risk	95% CI	
*FP use*	Intervention	Control					Lower	Upper
Male involvement in FP education	0.34	0.043	10.01	12.3	0.0000002*	0.29	0.23	0.34
Women’s education on FP	0.17	0.043	6.59	15.7	0.000006*	0.13	0.08	0.17
*Intention to use FP*								
Male involvement in FP education	1.59	1.29	5.14	19.7	0.00005*	0.3	0.17	0.42
Women’s education on FP	1.47	1.29	2.52	19.1	0.02*	0.18	0.03	0.31

^*^Significant at *p*-value < 0.05

Information on the intention to use FP shows that the difference of high intention to use FP and its 95% CI among the male involvement in FP education and women’s education on FP with control arm was 0.3 (0.17, 0.42) and 0.18 (0.03, 0.31), respectively. Thus, in the control arm the proportion of women who had high intention to FP use was 12.9% and AR increased about 3% among married women in the male involvement in FP education and 1.8% in the women’s education on FP arms (Table 4). Married women in the male involvement in FP education and women’s education on FP had higher odds ratio (OR) and its 95% CI of 11.4 (6.23, 20.93) and 4.6 (2.46, 8.71) more likely to use FP as compared with the control arm, respectively. Similarly, married women in the male involvement in FP education arm and women’s education on FP arm had 3.4 (2.48, 4.91) and 2.1 (1.50, 2.95) high intention to use of FP, respectively, as compared with the control arm.

### Any potential harms of the trials

The study report that there was no adverse eeffect following the provision of the interventions. Main reasons that contributed to this success were the following. Firstly, the decision for taking contraceptive mainly depend on the informed choice of the married women. Secondly, counselling to the married women on FP was provided by a trained health professional at healthcare facilities, and it included management of potential side effects and respective mitigation measures. Thirdly, a team that dealt with data monitoring safety was given responsibility for ensuring provision of the intervention based on the established protocols.

## Discussion

This study revealed that male involvement in FP education and women’s education on FP brings a significant change in increasing the rate of using and intention to use FP methods among the pastoralist communities. The number of participants who used and intended to use FP methods was higher in both intervention arms than the control arm. This finding was built on the previous finding that male involvement in FP education and women’s education on FP significantly increases modern FP use [18, 36, 37].

It’s also observed from the study that a FP intervention targeted merely at women and without involving males could not bring the desired change. The FP decision-making power of a husband was noticed to be a significant barrier for woman’s actual use of FP methods. Therefore, the role and influence of husbands need to be taken into account when developing an intervention about FP methods [36]. Also, male involvement in FP education helps to enhance in acceptance of a contraceptive and improves its practical use and continuation [37]. The provision of health education messages to males on FP could be one mechanism to improve male involvement in FP services. Importantly, in communities where married women are expected to adhere to vital cultural perspectives that promote having high number of children, using men as an entry point to the community would be vital. Proof of this, married women in this study are expected to respect and practice vital religious and cultural perspectives that hinder FP practices [11–13]. Indeed, all aspects of the pastoralist women’s health and well-being were strongly affected by religious perspective and belief [23] and the community believed that all health problems are caused by supernatural forces [38]. Such strong misconception and resistance on FP can be resolved with a continuous discussion with the influential groups such as husbands, and religious and clan leaders on the importance of FP. For instance, in this study, a religious leader educated men on FP concepts that do not contradict with Islam. This implies that involving religious leaders to teach men about FP services would help to have active male involvement in FP services. Active men involvement is reflected by such practices as discussing freely with their wives about FP, allowing them to exercise FP methods, accompanying them to health care facilities on FP matters, and providing them financial support for FP related purposes. Overall, the level of male involvement has been shown to influence women’s use of maternal health services, probably the most effective intervention in reducing maternal and infant mortality in the developing world by having optimal birth spacing, mitigating unwanted pregnancy and its consequences [39].

In general, and in pastoralist communities in particular, male involvement alone is not effective in promoting FP use, reducing high total fertility rate (TFR) and unmet need for FP. Women's education about FP also plays vital role in these aspects. Hence, promoting women's education about FP and, at the same time, engaging male in FP helps to enhance the health and well-being of mothers by reducing TFR and increasing FP use [1]. It also, improves the women’s knowledge, attitude and actual use of FP methods. Accordingly, a systematic review found that FP education to women can increase women’s knowledge of FP [24]. Similarly, other systematic review and meta-analysis on women's education on contraceptives found a significant increment of contraceptive use after providing education to women on FP [40]. Therefore, supporting women’s education on FP with good male involvement in FP services could be an excellent strategy to enhance FP use among the pastoralist communities.

A complete understanding of the determinants of women’s actual contraceptive behaviour is vital in preventing or minimizing behaviours that lead to unintended pregnancy by exploring their future intention to use FP [41]**.** This study found a significant increment in intention to use FP in the intervention arms following the provision of education on FP compared with the control arm. This was in line with studies done elsewhere [42, 43]. The effect could be explained by the fact that FP's good/high intention to use FP is an essential factor for women to use FP methods by considering having comprehensive knowledge and a positive attitude towards FP use. Intention to use of FP is a proximate determinant of actual use of FP [42]**.** A study in Ghana revealed that group versus individual FP counselling on FP does not bring a significant difference in intention to use FP [43]. The possible difference could be the difference in population, type of study design, and intervention given at the ground. In the Ghana study, a patient recovering on the gynecology ward and prepared for discharge, a randomized non-inferiority trial and individual vs group counselling was used as the source of population, type of the study design and the intervention done at the ground to counsel the eligible participants to enhance intention to use of FP, respectively.

Different studies had been done elsewhere in community settings to promote FP use. Such studies had employed different approaches to enhance FP practices [18, 19, 44–49]. This study used community-based structure (faema leaders) that has high acceptance in the community, a separate structure for both sexes and model individuals on FP practices. As a result, this study found a significant increment in use and intention to use FP. A study in rural India employed various intensive community mobilizations at different levels including women, spousal, family, community, and health system levels to enhance contraceptive uptake [19]. Also, Mali’s study included three groups: community-based contraceptive distribution (CBD), education only and controls arms. In the CBD, with the assistance of a local chief, each village was asked to select FP promoters (male and women). They were responsible for providing FP education via group meetings, home visits and selling contraceptives for the same sex. Intensive training was given for the community health agents and nurses to provide education only in the second arm, whereas no intervention was done in the control arm [44]. However, there was an interruption in the delivery of the intervention; it used service statistics, activity reports and interviews with trainers to monitor the progress of the intervention. Moreover, the intervention was given for 18 months [44]. In comparison with the Mali’s study, this study used the following strategies. Firstly, it used a faema leader to teach the community on the importance of FP by providing health education, not on selling the contraceptive. Secondly, the provision of health education message on FP was given with a regular schedule. Thirdly, to strengthen the health education message on FP a video-assisted message of role models or influential groups (married women who started FP use, men who were actively involved in FP services, religious leaders and local district’s FP experts) was used. Fourthly, a logbook was developed to monitor the progress of the intervention. Hence, approaching the community via their community members and using comprehensive community-based interventions would be vital to enhance FP use and intention to use FP.

A study done in rural India used a social cognitive theory (SCT) to guide an intervention for enhancing FP use [45]. Similarly, this study used an integrated behavioural model (IBM) to guide the provision of intervention at the ground. Guiding FP intervention with a behavioral model like IBM would help provide the intervention in the intended framework. The IBM is suitable in areas where the use and decision to use FP service is affected by different multifaceted factors (relatives, neighbours, husband, religious and clan leader). It is more comprehensive than earlier behavioral models (such as reasoned action and planned behaviour theory). Furthermore, it is capable of explaining a higher portion of the variance of the behavior (FP use and intention) under investigation. To enhance the predicting power of the model, an additional tool on women’s knowledge and male involvement in FP use was applied.

Conducting community-based interventions to promote FP use may not be carried out without facing a challenge. Different studies done elsewhere report different challenges in the provision of the intervention at the ground [44, 46, 49]. For instance, a study in rural Mali identifies the following challenges; community reluctance to accept FP message and use, insufficient funds to purchase contraceptives, contraceptive stock-out, inability to cover all the segment of the community in the provision of health education, religious influence with the concept of contraceptive use which is prohibited by Islam and fear of the community member to include their name in the promoter notebook. To overcome such challenges, increasing the number of the education sessions, arranging group talks during break time and reassuring the community members that their personal information would be managed confidentially by the promoters were used as a mitigation measures [44]. Similarly, a study in Kinshasa, DRC identified weak interaction with clinical services, having weak support and supervision of community-based distributors and recurring stock-outs of contraceptives as main challenges [46].

In line with these studies, this study had implemented the following mechanisms to address the anticipated challenges in providing community-based interventions on the ground. Firstly, a tailored message which has high acceptance by the community was discussed and developed. As a result, the emphasis of the health education message was on the importance of FP for spacing than limiting the number of children. Secondly, influential groups like religious leaders were approached and a consensus had been reached that educating FP does not contradict with Islam religion. Thirdly, to promote FP use and to strengthen the health education message on FP given by the faema leaders, a video recorded message of role models or influential groups (married women who started to use FP, men who were actively involved in FP services and local district’s FP experts) was used. Fourthly, adequate preparation was done for mobilizing the community members to attend the group FP education sessions. Fifthly, active engagement of the relevant stakeholders was undertaken from the very beginning so as to avail contraceptives on the spot, to have method mix in the health facilities, to counsel the women based on their informed consent and to provide FP services without fee. Sixthly, to reduce the work burden of the faema leader, an effort was made to focus them on the provision of health education on FP instead of selling contraceptives and collecting money (Additional files 1, 2, 3).

This study is novel in pastoralist communities where married women are nested with strong cultural and religious perspectives that promote a high number of children, poor women decision-making, and low FP coverage. As an approach, this study used the existing community-based structure like faema leaders to provide the health education message on FP. It used a cluster randomized controlled trial study design that is methodologically strong. The intervention was guided by a model (IBM). Finally, a reliable and valid tool that considers the pastoralist community's local context was developed [34].

The finding from the current study could be generalized to married women in a pastoralist community. However, the study has the following limitations. First, the evaluator of the outcome measures was not blinded to the type of intervention. Second, due to limited/small number of clusters per arm, the data was analyzed using a cluster-level summary analysis that did not account for covariate or baseline data adjustment in the final model. However, in the baseline data, there was no statistically significant difference in FP use and intention between the control arm and each intervention. Third, providing the intervention for nine months may be a short time to bring a huge change in FP use and intention. Fourth, even though CRT prefers to prevent contamination of information, a buffer zone was not employed. But the intervention cluster was separated from the control cluster by a distance of 20–40 km.

## Conclusion

The present study suggests that in such male-dominated pastoralist communities like the Afar region with significant religious barriers, community-based health education mainly targeting males appears to be a promising strategy for enhancing the practice of using and intending to use FP methods. Using the faema leaders to educate married women and men, use of video-assisted message, assisting the faema leaders using HEWs and health workers, and involving religious leaders in the FP education appears to increase the number of women who actually use and intend to use FP methods. However, further research could look at quantifying the contribution of each intervention in promoting FP use and intention to use. There is an urgent need to plan interventions through actively involving men in FP education and women’s education on FP for better FP use.

### Trial registration

The trial was registered in a ClinicalTrials.gov with a reference number of NCT03450564. And it can be accessed using https://clinicaltrials.gov/ct2/show/NCT03450564.

## Supplementary Information

**Additional file 1.** Data collecting instrument used in community-based interventions to promote family planning use among pastoralist women in Afar region, Ethiopia, 2020.

**Additional file 2.** Consort checklist used to report cluster randomized control trial of community-based interventions to promote family planning utilization among pastoralist women in the Afar region of Ethiopia.

**Additional file 3.** Data set from a cluster randomized control trial on the use of community-based interventions to promote family planning usage among pastoralist women in the Afar region of Ethiopia, 2020.

## Data Availability

To protect the participants’ anonymity, all the data was presented in the manuscript. In addition, the dataset was attached as additional supporting files to the scientific communities by removing all identifying/confidential client data.

## Abbreviations

AOR: Adjusted Odds Ratio
AMREF: Africa Medical Research Fund
CPR: Contraceptive Prevalence Rate
CRT: Cluster-Randomized Controlled Trial
DFID: Development for International Fund
RIF: Reproductive Innovative Fund
EDHS: Ethiopian Demographic Health Survey
FMOH: Federal Ministry of Health
FP: Family Planning
HSTP: Health Sector Transformation Plan
TFR: Total Fertility Rate
WHO: World Health Organization
