# Supplementary material for: Use of community-based interventions to promote family planning use among pastoralist women in Ethiopia: cluster randomized controlled trial
Source: BMC Womens Health. 2021 Aug 18;21:305. doi: 10.1186/s12905-021-01434-x (PMC8371816; doi:10.1186/s12905-021-01434-x)
Supplement: Supplementary file 1 — Additional file 1. Data collecting instrument used in community-based interventions to promote family planning use among pastoralist women in Afar region, Ethiopia, 2020. [file 12905_2021_1434_MOESM1_ESM.docx]

**Tools on family planning in the pastiarlist community**

| **Direct Measurement: Expressional attitude** | |
| --- | --- |
| Q1 | For me, using family planning to space or limit birth is …   1. Bad -2 -1 0 1 2 Good 2. Unpleasant -2 -1 0 1 2 Pleasant 3. Inconvenient -2 -1 0 1 2 Convenient 4. Worthless -2 -1 0 1 2 Useful 5. Unsatisfactory-2 -1 0 1 2 Satisfactory 6. Harmful -2 -1 0 1 2 Beneficial 7. Shameful -2 -1 0 1 2 Proud 8. Wastage of resource -2 -1 0 1 2 Productive 9. Source of Sickness -2 -1 0 1 2 Healthful 10. Weak -2 -1 0 1 2 Strong 11. Discouraging -2 -1 0 1 2 Rewarding 12. Sinful -2 -1 0 1 2 Goodness |
|  | **Direct Measurement: Instrumental attitude** |
| Q2 | For me using family planning makes me…   1. Deteriorate quality life -2 -1 0 1 2 Improve quality life 2. Weakened -2 -1 0 1 2 Empowered 3. Sad -2 -1 0 1 2 Happy 4. Sick -2 -1 0 1 2 Healthy |
|  | **Direct Measurement: Injective norm** |
| Q3 | How do you think other individuals in your community will evaluate family planning use   1. Lead to criticism -2 -1 0 1 2 Acceptable 2. Discouraging -2 -1 0 1 2 Rewarding 3. Refused -2 -1 0 1 2 Approved 4. Unwanted -2 -1 0 1 2 Wanted |
|  | **Direct measurement: Descriptive norm** |
| Q4 | How using family planning is common in your community?   1. Educated women -2 -1 0 1 2 Not educated 2. Urban resident -2 -1 0 1 2 rural residents 3. Being non married -2 -1 0 1 2Being married 4. Not at all -2 -1 0 1 2 Wives/relatives of clan leader 5. Not at all -2 -1 0 1 2Wives/relatives of religious leader 6. Not at all -2 -1 0 1 2Wives/relatives of kebele leader 7. Not at all -2 -1 0 1 2 Wives/relatives of volunteer 8. Not at all -2 -1 0 1 2 Among TBA/TBA only 9. Have small number of children -2 -1 0 1 2 Have many children 10. When she becomes (weak ) -2 -1 0 1 2 When she Becomes strong 11. Being older age -2 -1 0 1 2 Being young age 12. Being monogamous wife -2 -1 0 1 2 Being polygamous wife 13. Rarely -2 -1 0 1 2 common |
| Q5 | **Direct measurement: Perceived Behavioral Control (controllability)** |
|  | For me using family planning is…   - 1. Distant -2 -1 0 1 2 Nearly accessible   2. Religiously prohibited -2 -1 0 1 2 Religiously Allowed   3. Psychologically inconvenient -2 -1 0 1 2 Convenient   4. Depends on my husband decision -2 -1 0 1 2 Up to my decision   5. Not affordable -2 -1 0 1 2 Affordable |
|  | **Direct measurement: Self efficacy** |
| Q6 | For me using family planning is   1. Difficult -2 -1 0 1 2 Easy 2. Impossible -2 -1 0 1 2 Possible 3. Influenced by others -2 -1 0 1 2 Assertive 4. Hard to remind the schedule -2 -1 0 1 2 Easy to remind |

**Intention to use of Family Planning**

| S. N | Question | Response |
| --- | --- | --- |
| Q1 | At this moment, I can list some of the benefits of family planning use I would gain if I use it? | 1. Uncertain  2. Not sure  3.Certainly |
| Q2 | I am happy if I could use family planning to space the number of children, I would have in the future | 1.Disagree  2.Not sure  3.Agree |
| Q3 | I am happy if I could use family planning to limit the number of children, I would have in the future | 1.Disagree  2.Not sure  3.Agree |
| Q4 | I am willing to use family planning to space/limit number of children | 1.Disagree  2.Not sure  3.Agree |
| Q5 | I have already decided that I should use family planning in the near future. | 1.Disagree  2.Not sure  3.Agree |
| Q6 | I have ever used family planning in the previous 6 months and I found it relevant me. | 1. Uncertain  2. Not sure  3.Certainly |
| Q7 | I have ever used FP in the past 6 months and I am quite sure I will continue using it in the future. | 1. Uncertain  2. Not sure  3.Certainly |
| Q8 | It is expected that women in our community should use family planning and so do I | 1.Disagree  2.Not sure  3.Agree |

Perceived Male involvement

|  | **Level of women’s attitude towards Husband involvement:** *The following statements are positive statements that show the degree of your husband’s actual and potential/perceived involvement in FP. Please circle the number show your agreement:* **1(Disagree) 2(Not sure) 3(Agree)** in their order. | | | | | |
| --- | --- | --- | --- | --- | --- | --- |
| **If I want to use family planning ….** | | |  | | |  |
|  | | …My husband would discuss with me about the need to space childbirth. | 1 | 2 | 3 |  |
|  | | … My husband has ever discussed with me about the need to limit birth childbirth |  |  |  |  |
|  | | My husband would allow me to discuss with others or attend awareness creation activities on Family planning | 1 | 2 | 3 |  |
|  | | My husband would share me importance information about family planning | 1 | 2 | 3 |  |
|  | | For me convincing my husband to allow me to use family planning may be easy for me | 1 | 2 | 3 |  |
|  | | If I am going to use family planning, I think my husband will allow me to use it | 1 | 2 | 3 |  |
|  | | My husband has ever involved in the decision to use family planning | 1 | 2 | 3 |  |
|  | | My husband has ever participated in making the choice of the type of family planning | 1 | 2 | 3 |  |
|  | | My husband would handle the domestic activities to let me visit the the health facility for family planning | 1 | 2 | 3 |  |
|  | | My husband would provide me financial support to visit the the health facility for family planning | 1 | 2 | 3 |  |
|  | | My husband would accompany me to the health facility if I want to use family planning. | 1 | 2 | 3 |  |
|  | | My husband would remind me of the schedule for family planning not to forget it. | 1 | 2 | 3 |  |

**Expressional attitude**

| Q1 | How pleasant if you use family planning for limiting the number of children | 1. Unlikely 2. Not Sure 3. Likely |
| --- | --- | --- |
| Q2 | Does limiting the number of children has anything worthy for you? | 1. Worthless 2. Not sure 3. Worthy |
| Q3 | How pleasant you will be if you use family planning? | 1. Unlikely 2. Not Sure 3. Likely |
| Q4 | How important the pleasure you would get if you use family planning | 1. Not essential 2. Not sure 3. Essential |
| Q5 | How comfortable is it, if you use family planning | 1. On edge 2. Not Sure 3. Comfortable |
| Q6 | How the comfort of family planning methods matters for you? | 1. Not matter 2. Not sure 3. Highly |
| Q7 | How do you feel if you use family planning? | 1. Shameful 2. Not Sure 3. Proud |
| Q8 | How you are concerned with psychological factors following family planning use? | 1. Certainly 2. Not sure 3. Uncertain |
| Q9 | How would you feel if family planning methods administered through injection? | 1.Bad  2. Not sure  3. Good |
| Q10 | Does the pain following use of injectable methods bother you? | - 1. Definitely   2. Not Sure   3. Not at all |

**Instrumental attitude**

| Q1 | Do you believe that using modern contraceptives could space childbirth? | 1. Unlikely 2. Not sure 3. Likely |
| --- | --- | --- |
| Q2 | How do evaluate the benefit/s associated to spacing childbirth | 1. Harmful 2. Not sure 3. Beneficial |
| Q3 | Did you believe that frequent birth has an effect on the health of the mother? | 1. Uncertain 2. Not sure   Certain |
| Q4 | How giving many children is worthy of you? | 1. Worthless 2. Not sure 3. Worthy |
| Q5 | Do you believe that modern contraceptives could improve the health of the child? | 1. Unlikely 2. Not sure 3. Likely |
| Q6 | How frequently you bother about the health of your children? | - 1. Always   2. Not sure   3. Rarely |
| Q7 | Do you believe that family planning could limit the number of children? | 1. Unlikely 2. Not sur3\e 3. Likely |
| Q8 | How limiting the number of children is worthy for you | 1. Worthless 2. Not sure 3. Worthy |
| Q9 | Do you believe that modern contraceptives could improve the health of the mother? | 1. Unlikely 2. Not sure 3. Likely |
| Q10 | How you are wondering about your health associated with giving birth? | 1. Rarely 2. Not sure 3. Always |
| Q11 | Did you believe that frequent birth has an effect on the health of the child? | 1. Uncertain 2. Not sure 3. Certain |
| Q12 | How do evaluate having many children for you? | 1. Worthless 2. Not sure 3. Worthy |
| Q13 | Do you think modern contraceptive methods are effective to delay pregnancy? | 1. Unlikely 2. Not sure 3. Likely |
| Q14 | How do you evaluate the benefit of family planning for delaying pregnancy for you? | 1. Harmful 2. Not sure 3. Beneficial |
| Q15 | Does the use of family planning make the women healthy? | 1. Uncertain 2. Not sure 3. Certain |
| Q16 | How you are concerned with the health of women related to childbirth? | 1. Rarely 2. Not sure 3. Always |

**Indirect measurement: Subjective (Injunctive Descriptive) norm**

| S.N | Question | Response |
| --- | --- | --- |
| Q1 | Do you think that most of your close relatives use family planning? | 1.Uncertain  2.Not sure  3.Certain |
| Q2 | Does your close relatives’ status of FP use affect your willingness to use family planning? | 1.Uncertain  2.Not sure  3.Certain |
| Q3 | If you want to use FP, do you believe that your religious leader in your community would annoy it? | 1.Uncertain  2.Not sure  3.Certain |
| Q4 | Regarding with FP use, do you believe that you should always do what your religious leaders think | 1.Uncertain  2.Not sure  3.Certain |
| Q5 | Do you think your husband’s close relatives use family planning? | 1.Disagree  2.Not sure  3.Agree |
| Q6 | Does your husband’s close relatives’ status use family planning influence your use to family planning? | 1.Uncertain  2.Not sure  3.Certain |
| Q7 | If you want to use FP, do you believe that your clan leader in your community would approve it? | 1.Uncertain  2.Not sure  3.Certain |
| Q8 | Regarding FP use, do you believe that you should always do what your clan leaders think? | 1.Uncertain  2.Not sure  3.Certain |
| Q9 | If you want to use FP, do you believe that your grandparents would be happy with it? I | 1.Uncertain  2.Not sure  3.Certain |
| Q10 | Regarding with FP use, do you believe that you should always do what your parents think | 1.Uncertain  2.Not sure  3.Certain |
| Q11 | Do you think that wives of your religious leaders in your community use family planning? | 1.Disagree  2.Not sure  3.Agree |
| Q12 | Does the use of FP among wives of your religious leaders inhibit or affect your use to FP? | 1.Uncertain  2.Not sure  3.Certain |
| Q13 | If you want to use FP, do you believe that your husband’s ‘relatives would approve it? | 1.Uncertain  2.Not sure  3.Certain |
| Q14 | Regarding FP use, do you believe that you should always do what your husband’s relatives think? | 1.Uncertain  2.Not sure  3.Certain |
| Q15 | If you want to use FP, do you believe that your neighbors would blame you? | 1.Uncertain  2.Not sure  3.Certain |
| Q16 | Regarding FP use, do you believe that you should always do what your neighbors think? | 1.Uncertain  2.Not sure  3.Certain |
| Q17 | Do you think those wife/ wives of your clan leaders in your community use family planning? | 1.Disagree  2.Not sure  3.Agree |
| Q18 | Do you believe that the use of FP among wives of your clan leaders affects your use to FP? | 1.Uncertain  2.Not sure  3.Certain |
| Q19 | If you want to use FP, do you believe that your husband would allow you to practice it? | 1.Uncertain  2.Not sure  3.Certain |
| Q20 | Regarding FP use, do you believe that you should always do what your husband thinks? | 1.Uncertain  2.Not sure  3.Certain |
| Q21 | Do you think that most of the women in your neighbors use family planning? | 1.Disagree  2.Not sure  3.Agree |
| Q22 | Do your neighbors of practice affect your use of family planning? | 1.Uncertain  2.Not sure  3.Certain |

**Perceived control**

| S.N | **Question** | Response |
| --- | --- | --- |
| Q1 | Do you think that husbands in your community disallow their wives to use family planning? | 1.Agree  2.Not sure  3.Disagree |
| Q2 | How the disallowance of husbands in your community matter you to use family planning? | 1.Certain  2.Not sure  3.Uncertain |
| Q3 | Do you believe that opposition from others is likely to happen consequently to family planning use? | 1.Likely  2.Not sure  3.Unlikely |
| Q4 | Do you think that possible opposition from others could influence your decision to use family planning? | 1.Highly  2.Not sure  3.Does not matter |
| Q5 | Do you believe that side effect is likely to happen for you following family planning use? | 1.Likely  2.Not sure  3.Unlikely |
| Q6 | How possible side effect could matter for you to use family planning? | 1.Highly  2.Not sure  3.Does not matter |
| Q7 | Do you think is there is a risk of discrimination by community members if they know that you are using family planning? | 1.Agree  2.Not Sure  3.Disagree |
| Q8 | How possible discrimination could matter your practice to use family planning? | 1.Highly  2.Not sure  3.Does not matter |
| Q9 | Do you think that the cost of family planning may be expensive if you decided to use it? | 1.Certain  2.Not Sure  3.Uncertain |
| Q10 | Do you believe that the cost of family planning would worry you if you want to use it? | 1.Highly  2.Not sure  3.Does not matter |

**Self-efficacy**

| Q1 | If want to use family planning, I am certain that I would afford the cost of it. | 1.Disagree  2.Not sure  3.Agree |  |
| --- | --- | --- | --- |
| Q2 | If want to use family planning, I am confident that I always would keep the appointment regarding it. | 1.Disagree  2.Not sure  3.Agree |  |
| Q3 | Though I need to space childbirth, I am not sure that I always can get methods of my choice in the health facilities. | 1.Agree  2.Not sure  3Disagree |  |
| Q4 | If want to use family planning, I am confident to convince your husband that I should use it. | 1.Disagree  2.Not sure  3.Agree |  |
| Q5 | If I want to use family planning, I am confident that I can ask and discuss with health providers on how to use it. | 1.Disagree  2.Not sure  3.Agree |  |
| Q6 | If want to use family planning, I am certain that I would overcome opposition from others elsewhere. | 1.Disagree  2.Not sure  3.Agree |  |
| Q7 | It is up to me, If I want to use family planning, I can do it. | 1.Disagree  2.Not sure  3.Agree | |
